# Safety and Outcome of High-Flow Nasal Oxygen Therapy Outside ICU Setting in Hypoxemic Patients With COVID-19*

**DOI:** 10.1097/CCM.0000000000006068

**Published:** 2023-10-19

**Authors:** Matthijs L. Janssen, Yasemin Türk, Sara J. Baart, Wessel Hanselaar, Yaar Aga, Mariëlle van der Steen-Dieperink, Folkert J. van der Wal, Vera J. Versluijs, Rogier A.S. Hoek, Henrik Endeman, Dirk P. Boer, Oscar Hoiting, Jürgen Hoelters, Sefanja Achterberg, Susanne Stads, Roxane Heller-Baan, Alain V.F. Dubois, Jan H. Elderman, Evert-Jan Wils

**Affiliations:** 1 Department of Intensive Care, Franciscus Gasthuis and Vlietland Ziekenhuis, Rotterdam, The Netherlands.; 2 Department of Intensive Care, Erasmus MC, Rotterdam, The Netherlands.; 3 Department of Respiratory Medicine, Erasmus MC, Rotterdam, The Netherlands.; 4 Department of Respiratory Medicine, Franciscus Gasthuis and Vlietland Ziekenhuis, Rotterdam, The Netherlands.; 5 Department of Biostatistics, Erasmus MC, Rotterdam, The Netherlands.; 6 Department of Intensive Care, Martini Ziekenhuis, Groningen, The Netherlands.; 7 Department of Respiratory Medicine, Martini Ziekenhuis, Groningen, The Netherlands.; 8 Department of Intensive Care, Maasstad Ziekenhuis, Rotterdam, The Netherlands.; 9 Department of Intensive Care, Canisius-Wilhelmina Ziekenhuis, Nijmegen, The Netherlands.; 10 Department of Respiratory Medicine, Canisius-Wilhelmina Ziekenhuis, Nijmegen, The Netherlands.; 11 Department of Intensive Care, Haaglanden Medisch Centrum, Den Haag, The Netherlands.; 12 Department of Intensive Care, Ikazia Ziekenhuis, Rotterdam, The Netherlands.; 13 Department of Respiratory Medicine, Ikazia Ziekenhuis, Rotterdam, The Netherlands.; 14 Department of Respiratory Medicine, Admiraal de Ruyter Ziekenhuis, Goes, The Netherlands.; 15 Department of Intensive Care, IJsselland Ziekenhuis, Capelle aan den Ijssel, The Netherlands.; Department of Respiratory Medicine, IJsselland Ziekenhuis, Capelle aan den Ijssel, the Netherlands.; Department of Intensive Care, Admiraal de Ruyter Ziekenhuis, Goes, the Netherlands.; Department of Intensive Care, Haga Ziekenhuis, Den Haag, the Netherlands.; Department of Intensive Care, Erasmus MC, the Netherlands.; Department of Internal Medicine, Martini Ziekenhuis, Groningen, the Netherlands.; Department of Respiratory Medicine, Martini Ziekenhuis, Groningen, the Netherlands.

**Keywords:** COVID-19, high-flow nasal oxygen, hospital resource preservation, hypoxemia, intensive care unit, respiratory failure, safety

## Abstract

**OBJECTIVE:**

High-flow nasal oxygen (HFNO) therapy is frequently applied outside ICU setting in hypoxemic patients with COVID-19. However, safety concerns limit more widespread use. We aimed to assess the safety and clinical outcomes of initiation of HFNO therapy in COVID-19 on non-ICU wards.

**DESIGN:**

Prospective observational multicenter pragmatic study.

**SETTING:**

Respiratory wards and ICUs of 10 hospitals in The Netherlands.

**PATIENTS:**

Adult patients treated with HFNO for COVID-19-associated hypoxemia between December 2020 and July 2021 were included. Patients with treatment limitations were excluded from this analysis.

**INTERVENTIONS:**

None.

**MEASUREMENTS AND MAIN RESULTS:**

Outcomes included intubation and mortality rate, duration of hospital and ICU stay, severity of respiratory failure, and complications. Using propensity-matched analysis, we compared patients who initiated HFNO on the wards versus those in ICU. Six hundred eight patients were included, of whom 379 started HFNO on the ward and 229 in the ICU. The intubation rate in the matched cohort (*n* = 214 patients) was 53% and 60% in ward and ICU starters, respectively (*p* = 0.41). Mortality rates were comparable between groups (28-d [8% vs 13%], *p* = 0.28). ICU-free days were significantly higher in ward starters (21 vs 17 d, *p* < 0.001). No patient died before endotracheal intubation, and the severity of respiratory failure surrounding invasive ventilation and clinical outcomes did not differ between intubated ward and ICU starters (respiratory rate-oxygenation index 3.20 vs 3.38; Pao_2_:Fio_2_ ratio 65 vs 64 mm Hg; prone positioning after intubation 81 vs 78%; mortality rate 17 vs 25% and ventilator-free days at 28 d 15 vs 13 d, all *p* values > 0.05).

**CONCLUSIONS:**

In this large cohort of hypoxemic patients with COVID-19, initiation of HFNO outside the ICU was safe, and clinical outcomes were similar to initiation in the ICU. Furthermore, the initiation of HFNO on wards saved time in ICU without excess mortality or complicated course. Our results indicate that HFNO initiation outside ICU should be further explored in other hypoxemic diseases and clinical settings aiming to preserve ICU capacity and healthcare costs.

**KEY POINTS**

**Question**: To evaluate the safety and clinical outcomes of the application of high-flow nasal oxygen (HFNO) outside the ICU setting in patients with COVID-19.

**Findings**: In this prospective observational study of 608 hypoxemic adult patients with COVID-19, we found that initial HFNO application outside the ICU setting was not associated with excess intubation or mortality rate, or more complicated clinical course. Furthermore, such a strategy significantly saves time in the ICU.

**Meaning**: Outside the ICU HFNO initiation appears safe and may spare ICU resources, and such a strategy should be further explored in other hypoxemic diseases and clinical settings.

Hypoxemia is the main reason for hospitalization of patients with COVID-19 (1). A large proportion of these patients are subsequently admitted into the ICU and invasively ventilated due to progressive respiratory failure (1–4). Noninvasive respiratory support, such as high-flow nasal oxygen (HFNO) and noninvasive ventilation (NIV) have been shown to reduce respiratory effort and intubation rates in non-COVID-19 settings (5, 6). These strategies have been widely applied during the COVID-19 pandemic with the aim to reduce the risk of invasive mechanical ventilation (IMV) (7–12). Randomized clinical studies indicate that HFNO as compared with conventional oxygen therapy (COT) reduces intubation rates but does not affect mortality (13–16). These studies were predominantly performed within the ICU setting. To limit ICU referral in times of limited ICU resources, HFNO for hypoxemic failure has widely been applied in non-ICU settings (17–20). However, the safety of HFNO in non-ICU settings is unclear, as few studies have investigated the safety and outcome of HFNO application in non-ICU settings (12, 17, 21–23).

In this multicentre prospective observational study, we aimed to assess the safety and outcome of HFNO application outside the ICU setting in patients with COVID-19-associated acute hypoxemic failure.

## MATERIALS AND METHODS

### Objectives and Clinical Endpoints

The objective was to evaluate the safety and potential harm of application of HFNO in the non-ICU setting compared with ICU setting in patients with COVID-19-associated hypoxemic respiratory failure. The primary outcome was the intubation rate. Secondary outcomes with corresponding defined endpoints are listed in **Table 1**.

**TABLE 1.** Outcomes and Corresponding Endpoints

Outcomes	Endpoint(s)
Intubation (primary)	Intubation rate
Survival	In-hospital mortality
	28-d and 90-d mortality
ICU admission	Proportion of patients transferred to ICU
Time course during hospitalization	Duration of HFNO therapy
	Referral to ICU within 4 and 6 hr after HFNO start outside ICU
	Intubation within 2 and 4 hr after ICU admission
	Ventilator-free days at day 28 and 60 after HFNO initiation
	ICU-free days at day 28 and 60 after HFNO initiation
	Hospital-free days at day 28 and 60 after admission
Severity of respiratory failure before/after intubation	Spo_2_, respiratory rate, S/F ratio, P/F ratio, respiratory rate-oxygenation index, Paco_2_ before intubation
	Prone positioning after intubation
Complications during or after HFNO therapy	Pneumothorax/-mediastinum during HFNO, facial decubitus during HFNO intolerance for HFNO due to discomfort
	Preintubation mortality
	Severe complications during intubation, defined as cardiopulmonary resuscitation, deep desaturation (Spo_2_ < 80%) during intubation^a^, hypotension (systolic blood pressure < 90 mm Hg) during intubation^a^

HFNO = high-flow nasal oxygen, S/F ratio = SpO_2_/Fio_2_ ratio, P/F ratio = Pao_2_/Fio_2_ ratio.

a Data scored only in one center.

### Ethics and Approvals

This study was approved by the medical research ethics committee (Medical Research Ethics Committees United, MEC-U number W20.283), which waived the need for written informed consent due to the observational character of the study. However, some of the participating hospitals did obtain written informed consent if deemed necessary by local guidelines of institutional research committees. The study was registered on November 27, 2020, in the Dutch Trial Registry (LTR, NL9067). The study has been carried out in accordance with the Helsinki Declaration for medical research involving humans (24). Before the start of the study analysis, we decided to include a more in-depth safety evaluation as originally preplanned and stated in the trial registry. Hence several endpoints were added to the analysis. The study design and setting were unchanged. This report follows the STrengthening the Reporting of OBservational studies in Epidemiology (STROBE) guidelines for cohort studies; The STROBE checklist of this study is depicted in **Supplemental digital content** (http://links.lww.com/CCM/H429) (25).

### Study Design and Data Sources

We conducted a pragmatic prospective observational cohort study in 10 hospitals in The Netherlands. Local clinical protocols of participating hospitals were collected for medical treatment of COVID-19, the use of HFNO inside and outside ICU, nurse-to-patient ratio, and level of patient monitoring. Clinical decisions on do-not-intubate (DNI) orders, initiation of HFNO, transfer to ICU, or escalation of respiratory support were left at the discretion of the treating physicians. Clinical data (demographics, laboratory, treatment, and outcomes), including disease severity (Sequential Organ Failure Assessment [SOFA]- [26], 4C mortality- [27], and 4C deterioration score [28]) and respiratory parameters (Pao_2_/Fio_2_ [P/F] ratio, Spo_2_/Fio_2_ [S/F] ratio, and respiratory rate-oxygenation (ROX) index [29]) were prospectively collected at prespecified time points (**Fig. S1**, http://links.lww.com/CCM/H429) using standardized electronic forms (CASTOR eClinical data management platform). Data monitoring and quality control were performed by the local institution.

### Study Population

Adult patients hospitalized for acute hypoxemic respiratory failure due to polymerase chain reaction-proven infection with severe acute respiratory syndrome coronavirus 2 (SARS-CoV-2) were included. In this study patients with and without a DNI order were included. The analyses in this article are limited to patients without a DNI order. Patients were excluded if: 1) HFNO was used only as respiratory support peri-intubation or postextubation (i.e., the decision to intubate was made before HFNO initiation) or 2) HFNO was contra-indicated due to inappropriate fitting of the interface (such as recent upper airway surgery or anatomic variations), or 3) direct intubation was required, as clinically indicated. Patient’s follow-up continued up to 90 days after HFNO initiation.

### Statistical Analysis

Continuous variables were reported as median values with interquartile range (IQR). Categorical variables were reported as numbers with percentages. Differences between groups regarding continuous variables were analyzed by the Mann-Whitney *U* test. Differences between groups regarding categorical variables were analyzed using the Chi-square test or with Fisher exact test in the case of a cell with less than five subjects.

To compare outcomes between patients who initiated HFNO outside (ward starters) or in ICU (ICU starters) while adjusting for confounders, propensity score matching was performed. Using a logistic regression model with clinically important characteristics (consisting of demographic, respiratory, and laboratory variables listed in **Table S1**, http://links.lww.com/CCM/H429), a propensity score was estimated on the likelihood to be in either group (ward or ICU starter). Single imputation was used to impute missing values in the covariates under the assumption of missing at random. Nearest neighbor matching (1:1) with a caliper of 0.1 was used to match patients who initiated HFNO outside ICU with patients who initiated HFNO in ICU. Differences between matched patients regarding continuous variables were analyzed by Wilcoxon signed rank test, and in categorical variables, a McNemar test was used.

To compare the course of hospital admission between patients who started HFNO outside ICU and in ICU, we calculated hospital-free days since admission and ICU-free days, ventilator-free days and respiratory support-free days at days 28 and 60 from HFNO initiation as described elsewhere (30). To account for the competing risk of death, deceased patients were considered to have zero hospital-, ICU-, or ventilator-free days.

To determine predictors for ICU admission among ward starters a multivariable logistic regression analysis was performed on the same candidate predictor parameters as used for the propensity score matching. These candidate predictors were added without forward or backward selection. Outcomes of the multivariable logistic regression are represented by odds ratio (OR) with 95% CI.

The sample size was calculated for the primary outcome (intubation rate). Based on an assumed event rate of 55 per 100 patients (31), a minimum of 600 patients was required to assess the intubation rate with a margin of error below 4%.

The analyses were performed with R version 4.1.0. The MatchIT package was used to perform the propensity score matching. Two-sided *p* values less than 0.05 were considered statistically significant.

## RESULTS

### Clinical Protocols

All participating hospitals used HFNO in ICU; seven hospitals used HFNO in patients admitted to the non-ICU ward. Two hospitals used HFNO at the ward only after an initial trial of HFNO in ICU (these patients were considered as ICU starters). None of the centers used NIV or continuous positive airway pressure after HFNO failure in ward or ICU. The nurse-to-patient ratio on non-ICU wards facilitating HFNO start ranged from 1:3 to 1:4 during daytime and from 1:4.5 to 1:6.6 during night time. Physician presence on wards was generally 1 resident present per 10 patients during daytime and one on call for the whole ward during night time. Overall, one consultant was on call during day and night time. Four of seven centers used continuous monitoring of Spo_2_ and respiratory rate on the ward. Detailed descriptions of local clinical practice on HFNO and staffing in and outside and ICU are described in **Tables S2** and **S3** (http://links.lww.com/CCM/H429). Local protocols for COVID-19-specific treatment are depicted in **Table S4** (http://links.lww.com/CCM/H429).

### Total Cohort

**Figure 1.**
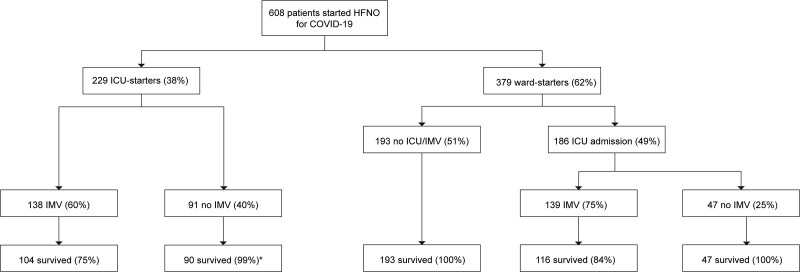
Flowchart for included patients with COVID-19 on high-flow nasal oxygen (HFNO). Survival percentages refer to the subgroup to which patients belong (i.e., ward starter or ICU starter, intubated or not intubated). *One patient died a week after ICU discharge due to severe pulmonary embolism, after initial respiratory recovery and successful weaning from HFNO. IMV = invasive mechanical ventilation.

Between December 1, 2020, and July 1, 2021, 608 patients were included, corresponding with the second and third COVID-19 waves in The Netherlands, during which the SARS-CoV-2 alpha variant was dominant. Follow-up was completed at 90 days in 510 of 608 patients (83.9%), 41 patients (6.7%) were lost to follow-up. Demographic and clinical data are presented in **Table 2** and **Table S5** (http://links.lww.com/CCM/H429). The majority of patients received dexamethasone (601/608, 98.8%) and 389 of 608 patients (64.0%) received an interleukin-6 receptor antagonist. Thirty-three patients (4.8%) had received at least 1 SARS-CoV-2 vaccination dose before admission. Of 608 patients, 415 (68.3%) were admitted to ICU during their hospital stay, of which 277 (45.5%) were intubated (**Fig. 1**). The in-hospital mortality rate was 9.5% (58/608); of note, none of the patients died before an intubation event. Cardiopulmonary resuscitation during intubation occurred in only one patient. Intolerance for HFNO was reported in 12 patients (2.0%). HFNO was complicated by pneumothorax/-mediastinum in five patients (0.8%) and facial decubitus was reported in one patient.

**TABLE 2.** Characteristics of the Study Cohort

	Overall (*n* = 608)	Ward Starters (*n* = 379)	ICU Starters (*n* = 229)	*p*
Age	61 (53–68)	61 (53–68)	61 (52–67)	0.61
Male sex	417 (68)	247 (65)	170 (74)	0.03
Obesity	252 (41)	146 (39)	106 (46)	0.67
Number of comorbidities				0.63
0	319 (52)	213 (56)	106 (46)	
1	178 (29)	105 (28)	73 (32)	
≥ 2	110 (18)	61 (16)	49 (21)	
Sequential Organ Failure Assessment score at hospital admission	3 (2–3)	2 (2–3)	3 (3–3)	< 0.001
4C mortality score at hospital admission	10 (7–12)	9 (7–12)	10 (8–12)	0.02
Variables before HFNO start				
S/F ratio	123 (116–204)	191 (123–220)	116 (113–120)	< 0.001
Fio_2_	0.80 (0.45–0.80)	0.60 (0.41–0.80)	0.80 (0.80–0.80)	< 0.001
Respiratory rate (/min)	28 (24–32)	26 (24–31)	30 (25–35)	< 0.001
Respiratory rate-oxygenation index	5.1 (3.9–7.5)	6.5 (5.0–8.5)	3.9 (3.4–4.7)	< 0.001
Outcomes				
Duration of symptoms at hospital admission (d)	8 (7–10)	8 (7–10)	9 (7–10)	0.24
Time between hospital admission and start HFNO (hr)	17 (2–47)	18 (3–43)	16 (2–59)	0.33
Intubation	277 (46)	139 (37)	138 (60)	< 0.001
Intubation ≤ 4 hr after HFNO start	28 (5)	9 (2)	19 (8)	0.001
Intubation ≤ 2 hr after ICU admission	43 (7)	36 (10)	7 (3)	< 0.01
Mortality before intubation	0	0	0	NA
In-hospital mortality	58 (10)	23 (6)	35 (15)	< 0.001
28-d mortality after HFNO start	38 (6)	13 (3)	25 (11)	< 0.001
Hospital-free days at day 28 after hospital admission (d)	13 (0–19)	15 (3–19)	6 (0–17)	< 0.001
ICU-free days at day 28 after HFNO start (d)	23 (11–28)	28 (17–28)	18 (0–23)	< 0.001
Ventilator-free days at day 28 after HFNO start (d)	28 (16–28)	28 (21–28)	22 (7–28)	< 0.001

CRP = C-reactive protein, HFNO = high-flow nasal oxygen, S/F ratio = Spo_2_/Fio_2_ ratio.

Categorical variables are presented as numbers with percentages, continuous variables are presented as medians with interquartile range. Differences between groups regarding continuous variables were analyzed with the Mann-Whitney *U* test. Differences between groups regarding categorical variables were analyzed using the χ^2^, or with Fisher exact test in case of a cell with less than five subjects.

### Ward Versus ICU Starters

First, we compared the characteristics and outcomes between the ward and ICU starters in a nonpropensity-matched analysis. HFNO was started on the ward in 379 of 608 patients (ward starters; 62.3%) and in 229 in ICU (ICU starters, 37.7%, Fig. 1). Hypoxemia at HFNO initiation was more severe in ICU starters as compared with ward starters (Table 2). One-hundred eighty-six of 379 ward starters (49%) were transferred to ICU during admission. Of 379 ward starters, 139 (36.7%) were intubated compared with 138 of 229 (60.3%; *p* < 0.001) among ICU starters. In-hospital mortality was higher among ICU starters. The number of hospital-free days, ICU-free days, and ventilator-free days were lower in ICU starters compared with ward starters (Table 2).

### Propensity-Matched Cohort: Ward Versus ICU Starters

ICU starters were more severely ill than ward starters, as illustrated by more severe hypoxemia, and higher SOFA and 4C mortality scores (Table 2). Therefore, propensity score matching was applied. One-hundred seven ward starters were matched to ICU starters. After matching, baseline characteristics, respiratory parameters at HFNO initiation, originating centers, and propensity scores were equally distributed (**Tables S6** and **S7**, http://links.lww.com/CCM/H429). Baseline characteristics and outcomes comparing matched and nonmatched patients are depicted in **Tables S8** and **S9**, http://links.lww.com/CCM/H429).

**Figure 2.**
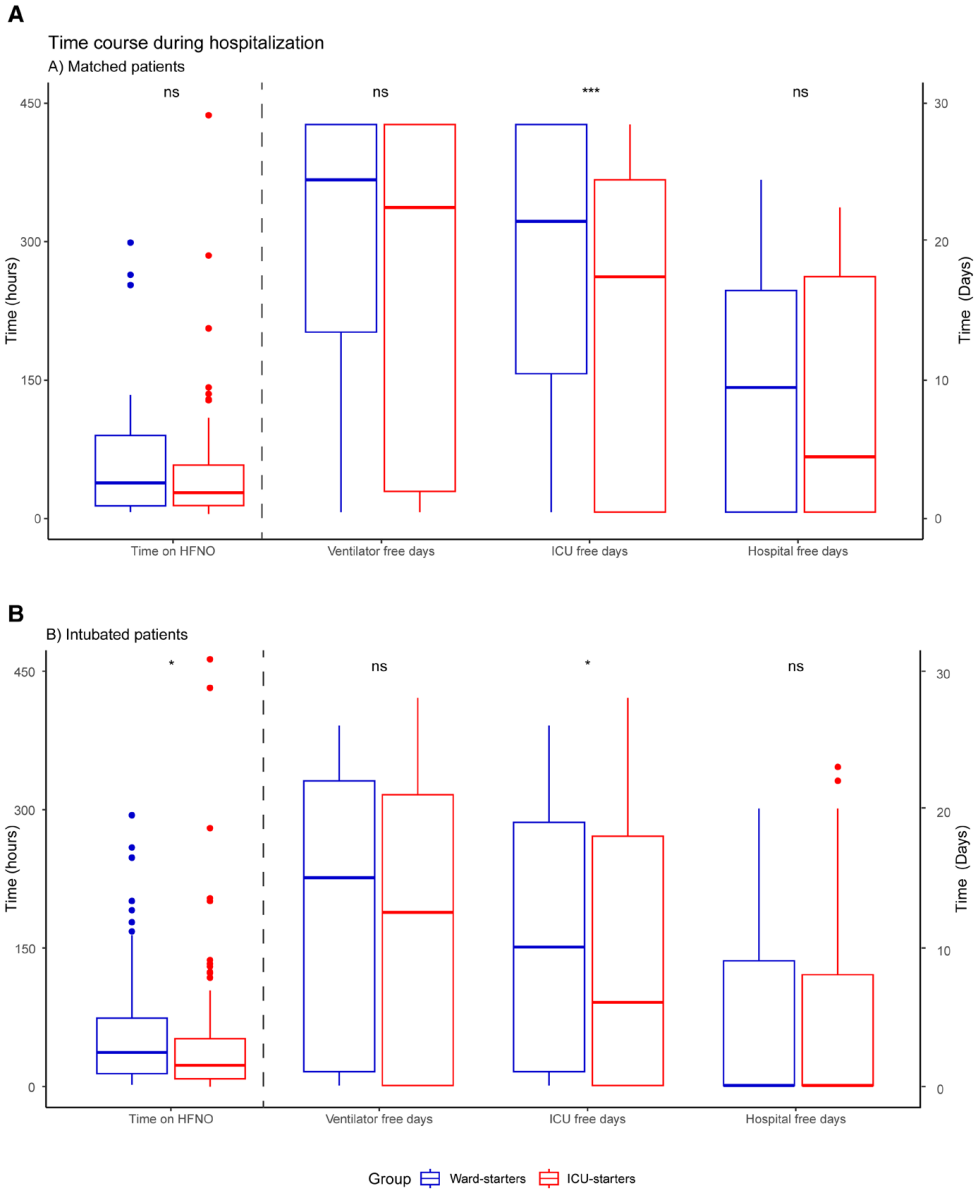
Time course during hospitalization, compared between ward and ICU starters. Results are separated for the propensity-matched cohort (**A**) and intubated patients (**B**). Time on high-flow nasal oxygen (HFNO) represents the time between HFNO initiation and intubation in both figures. One asterisk represents *p* value below 0.05, three asterisks represent *p* values below 0.001. ns = nonsignificant.

Sixty-eight of 107 matched ward starters (63.3%) were referred to ICU during their disease course (**Table 3**; and Table S6, http://links.lww.com/CCM/H429). Fifty-seven (53.3%) were intubated, compared with 64 of 107 matched ICU starters (59.8%; *p* = 0.42). Mortality rates, hospital-free days, and ventilator-free days were similar for matched ward and ICU starters (Table 3 and **Fig. 2**). The number of ICU-free days was higher for ward starters compared with ICU-starters (median difference of 4 d for ICU-free days at day 28 (*p* < 0.001)) and at day 60 (*p* = 0.02, Fig. 2). The time between HFNO initiation and intubation was similar between the groups (37.3 [9.3–85.5] vs 23.7 [9.0–52.6] hr; *p* = 0.17).

**TABLE 3.** Outcomes in the Propensity-Matched Ward Versus ICU Starters

	Ward Starters (*n* = 107)	ICU Starters (*n* = 107)	*p*
Duration of symptoms at hospital admission (d)	8 (6–10)	9 (7–10)	0.25
Time between hospital admission and start HFNO (hr)	5 (2–59)	16 (2–67)	0.69
Time on HFNO until ICU admission (hr)	16 (4–43)	Not applicable	NA
Time on HFNO until intubation (hr)	37 (9–86)	24 (9–53)	0.17
Time between ICU admission and intubation (hr)	5 (2–23)	23.5 (10–51)	0.01
ICU admission	68 (64)	107 (100)	NA
Intubation	57 (53)	64 (60)	0.42
Intubation ≤ 4 hr after HFNO start	7 (7)	7 (7)	1.00
Intubation ≤ 2 hr after ICU admission	16 (15)	1 (1)	0.001
Mortality before intubation	0	0	NA
Prone positioning after intubation	47 (44)	51 (48)	0.50
In-hospital mortality	14 (13)	19 (18)	0.45
28-d mortality after HFNO initiation	8 (8)	13 (13)	0.24
Hospital-free days at day 28 after hospital admission (d)	9 (0–16)	4 (0–17)	0.62
ICU-free days at day 28 after HFNO start (d)	21 (10–28)	17 (0–24)	< 0.001
Ventilator-free days at day 28 after HFNO start (d)	24 (13–28)	22 (2–28)	0.13

HFNO = high-flow nasal oxygen.

Categorical variables are presented as numbers with percentages, continuous variables are presented as medians with interquartile range. Differences between groups regarding continuous variables were analyzed by Wilcoxon signed rank. Differences between groups regarding categorical variables were analyzed using the McNemar test.

### Intubated Patients

To explore whether a course of HFNO outside ICU is associated with a worse clinical status before intubation and negatively affects outcome, we next compared outcomes between intubated ward and ICU starters (**Table 4**). Baseline characteristics were similar for intubated ward and ICU starters (**Table S10**, http://links.lww.com/CCM/H429). The severity of hypoxemia before intubation was similar in ward and ICU starters (P/F ratio [65 (58–75) vs 64 (56–73) mm Hg; *p* = 0.52] and ROX index [3.2 (2.8–3.8) vs 3.4 (2.7–4.0), *p* = 0.41]. The proportion of patients undergoing prone positioning after intubation was similar for the groups (112 [80.6%] vs 104 [78.2%]; *p* = 0.74). The initiating center recorded complications during 105 intubation events: hypotension occurred in 10 (9.5%) and severe hypoxemia in 16 (15.2%) of patients, with no statistically significant differences between ward and ICU starters.

**TABLE 4.** Characteristics and Outcomes in Intubated Ward Versus ICU Starters

	Ward Starters (*n* = 139)	ICU Starters (*n* = 138)	*p*
Variables on HFNO before intubation			
S/F ratio	95 (91–104)	93 (90–100)	0.03
P/F ratio	65 (58–75)	64 (56–73)	0.52
Paco_2_ (mm Hg)	34 (31–37)	35 (33–39)	< 0.01
Respiratory rate (/min)	30 (26–35)	30 (24–35)	0.26
Respiratory rate-oxygenation index	3.2 (2.8–3.8)	3.4 (2.7–4.0)	0.41
Outcomes			
Duration of symptoms at hospital admission (d)	7 (6–9)	8 (7–10)	0.04
Time between hospital admission and start HFNO (hr)	18 (3–48)	16.5 (2–65)	0.52
Time on HFNO until ICU admission (hr)	18.5 (5–41)	NA	NA
Time on HFNO until intubation (hr)	37 (14–74)	23 (9–51)	0.01
Time between ICU admission and intubation (hr)	6 (2–22)	23 (9–51)	< 0.001
Intubation ≤ 4 hr after HFNO initiation	9 (7)	19 (14)	0.05
Intubation ≤ 2 hr after ICU admission	36 (26)	7 (5)	< 0.001
Cardiopulmonary resuscitation during intubation	0 (0)	1 (0.7)	NA
Prone positioning after intubation	112 (81)	104 (78)	0.74
In-hospital mortality	23 (17)	33 (25)	0.13
28-d mortality after HFNO initiation	13 (9)	24 (17)	0.06
Hospital-free days at day 28 after hospital admission (d)	0 (0–9)	0 (0–8)	0.92
ICU-free days at day 28 after HFNO start (d)	10 (1–19)	6 (0–18)	0.04
Ventilator-free days at day 28 after HFNO start (d)	15 (1–22)	12.5 (0–21)	0.19

HFNO = high-flow nasal oxygen, S/F ratio = Spo_2_/Fio_2_ ratio, P/F ratio = Pao_2_/Fio_2_ ratio.

Categorical variables are presented as number with percentages, continuous variables are presented as medians with interquartile range. Differences between groups regarding continuous variables were analyzed with the Mann-Whitney *U* test. Differences between groups regarding categorical variables were analyzed using the χ^2^, or with Fisher exact test in case of a cell with less than five subjects.

The number of ICU-free days at 28 days after HFNO initiation were significantly higher for HFNO ward starters compared with ICU starters (10 d [1–19] vs 6 d [0–18]; *p* = 0.04, Fig. 2). Mortality rates, and the number of hospital- and ventilator-free days did not differ between groups.

### Predictors for ICU Admission in Ward Starters

Finally, we explored which clinical predictors in ward starters were associated with ICU admission. Results of the multivariable logistic regression analysis on predictors for ICU admission among patients at the start of HFNO outside ICU are depicted in **Table S11** (http://links.lww.com/CCM/H429). Higher respiratory rate (OR 1.09) and higher oxygen administration before HFNO start (air-entrainment or nonrebreathing mask < 15 L/min [OR 3.35]; nonrebreathing mask 15 L/min [OR 3.57]) were associated with ICU admission. Higher oxygen saturation (OR 0.87) and longer duration of symptoms at HFNO initiation (OR 0.88) were associated with a lower risk of ICU admission.

## DISCUSSION

In this multicentre cohort study, we compared the safety and outcome between initiation of HFNO in non-ICU versus ICU settings in patients with acute hypoxemia due to COVID-19. We observed that patients who started HFNO on the ward had comparable intubation and mortality rates as their matched ICU counterparts, but their number of ICU-free days was higher. Furthermore, safety outcomes were similar. No signal of harm was observed in patients who were eventually intubated with a precourse of HFNO on the ward. Finally, exploratory analysis suggests that the risk for ICU admission among patients who started HFNO outside ICU was higher with shorter duration of symptoms, higher respiratory rate, lower oxygen saturation, and higher level of oxygen administration before HFNO start.

Recent randomized controlled trials indicate that HFNO as compared with conventional oxygen decreases intubation rates but does not affect mortality (14–16, 32, 33). These studies included patients with comparable disease severity as the present study, but the number of patients with HFNO initiated on wards was limited (15, 16, 32, 33). Before the COVID-19 pandemic, a few observational studies provided exploratory data on the use of HFNO outside ICU (18, 34). The COVID-19 pandemic was accompanied by widespread use of HFNO on wards (14, 17, 19, 22). Earlier randomized and observational studies on HFNO focused on clinical rather than safety outcomes, included a significant number of patients with treatment restrictions, and did not compare HFNO initiation outside versus in ICU (15–17, 19, 22, 32, 33, 35, 36). In contrast, our pragmatic observational study is the largest and most elaborate study to date that assessed in-depth safety outcomes combined with clinical outcomes and detailed in-hospital time courses. Only hypoxemic patients without treatment restrictions were included and practice variation in the participating centers enabled a propensity-matched comparison between ward and ICU starters.

Insufficient monitoring, limited availability of skilled personnel and equipment, and delayed escalation to IMV have been major concerns that limit the use of HFNO outside ICU (37, 38). The wards with HFNO capacity in this study were frequently equipped with continuous monitoring, had a daytime nurse-to-patient ratio of 1:3–1:4, had access to contemporary pharmacologic COVID-19 therapies, and resources to escalate respiratory support were available around the clock. These circumstances are in line with pulmonary intermediate care units nested in regular respiratory wards as predominantly introduced in high-income countries but in contrast with those in low- or middle-income countries (17, 39, 40). Within this context, we observed no deaths before an intubation event. In contrast, previous studies performed during the height of the pandemic reported relevant numbers of full-code patients who succumbed before an intubation attempt while on HFNO, both inside and outside the ICU. Suggested contributing factors were limited monitoring, low nurse-to-patient ratios, and shortage of ICU capacity (17, 22).

Our data show that initiation of HFNO on wards significantly increased the number of ICU-free days, without the cost of excess mortality and morbidity. This implies that utilization of HFNO on wards reduces demand for ICU, saves healthcare costs, and preserves ICU capacity for patients in need of high-intensity care. Previous studies have suggested that HFNO outside the ICU may reduce the demand for ICU services (17, 19, 22, 35, 36). These exploratory studies were however hampered by methodological drawbacks such as single-center study designs, small sample sizes, heterogeneous populations, and importantly the lack of an appropriate comparator. In line with our observations but within the ICU setting, Mellado-Artigas et al (41) also observed a reduction in duration of ICU stay when HFNO was applied and compared with early initiation of IMV. Strategies to spare ICU resources have an impact on healthcare costs, but may also help to prioritize resources to other patients. This becomes ever more relevant as the current and future demand for ICU beds will likely grow, irrespective of a new pandemic (42). Our results and the experience gained during the COVID-19 pandemic may further serve as an invitation to more rigorously assess whether other hypoxemic diseases are amendable to outside ICU strategies (43). Furthermore, given the potential long-term sequelae for patients and their relatives related to an ICU admission, keeping patients on the ward may also be beneficial from a patient perspective (44, 45).

Using propensity score matching, we compared the more severely affected ward starters to a representative sample of matched ICU starters. Applying HFNO in patients with less severe oxygenation failure, such as the nonmatched ward starters in our study remains questionable. Crimi et al (14) recently showed that HFNO in patients with COVID-19 and mild-to-moderate hypoxemia was not superior to COT. Scarcity in evidence-based directions on when to initiate HFNO and clear-cut criteria for when to escalate respiratory support to IMV remains an urgent challenge in HFNO therapy. A prolonged course of HFNO and delayed escalation of respiratory support are postulated to induce patient-self-inflicted lung injury (P-SILI), but its relevance remains a matter of intense debate (46–48). P-SILI is anticipated to result in a more severe disease course, that may present clinically as more severe hypoxemia, longer duration of IMV, and ultimately high mortality rates. In our study, intubated patients who started HFNO outside ICU were on HFNO for a longer period than their ICU counterparts. A longer period of HFNO was not associated with more severe oxygenation failure before intubation, a higher proportion of complicated intubations or prone positioning, less ventilator-free days, or higher mortality rate. Irrespective of the existence of P-SILI, our data suggest that the ward-HFNO strategy, including a longer period of preintubation HFNO did no further harm.

To improve clinical decision-making when applying HFNO in the non-ICU setting, identification of early predictors of ICU admission may provide such guidance. Our exploratory analysis suggests that the risk for ICU admission was higher with shorter duration of symptoms, higher respiratory rate, lower oxygen saturation, and higher level of oxygen administration before HFNO start. More elaborate analyses are underway also taking into account the response to HFNO therapy and comparing these predictors with others, such as the combined ROX index (49).

Our study comes with strengths and limitations. Major strengths of our study are its large sample size, its prospective and multicenter character, and the high level of completeness of detailed data on the hospital course of patients with severe COVID-19. We acknowledge several limitations. First, observational studies are hampered by residual and unmeasured confounding in contrast to randomized studies. As randomizing patients between ICU and ward was not deemed feasible, we used propensity score matching to assemble comparable groups. We retained a representative sample matched for variables likely to affect both the exposure and outcome of interest (50). Second, due to the pragmatic study design, HFNO was initiated within a wide range of hypoxemic respiratory failure severity and at different locations in the participating centers. Although our analysis suggests that a large part of hypoxemic patients can be safely treated on the ward, what to do best for an individual patient remains to be studied. Risk stratification may further add guidance on which patient may benefit (most) from HFNO and early ICU admission (51). Importantly, no inferences can be made for patients with hypercapnic respiratory failure, as only patients with hypoxemic respiratory failure were included in our study. Third, our study was performed in the period when the alpha SARS-CoV-2 variant was dominant and vaccination coverage was low. Nonetheless, it is likely that our results remain relevant as the disease course of currently hospitalized patients with hypoxemic COVID-19 does not appear to vary greatly for consecutive SARS-CoV-2 variants (52, 53). Furthermore, contemporary treatment regimens were already in place during the study period, including dexamethasone and interleukin-6 receptor antagonists. Fourth, the acute burden of COVID-19 is currently low with manageable numbers of hospitalized hypoxemic patients. New outbreaks of SARS-CoV-2 or other respiratory viruses are nonetheless foreseen. Fifth, we recognize the difficulty in generalizing our results to healthcare systems with different patient populations and limited resources. Our data however suggest that HFNO on wards is a viable option that can be weighted against the possibilities for monitoring, escalation of respiratory therapy, and the patient population that presents itself locally.

## CONCLUSIONS

In conclusion, the application of HFNO outside the ICU setting in hypoxemic patients with COVID-19 appears safe and may help to use the finite ICU capacity for patients in high need of this costly care. Furthermore, a precourse of HFNO on the ward compared with immediate ICU admission was not associated with clinical harm, even for those eventually intubated. Our findings may help clinicians in deciding on a trial of HFNO outside ICU, taking into account patient selection and resources available within their own healthcare system.

## ACKNOWLEDGMENTS

We express our gratitude for their contributions to this study J.C.C.M. in ’t Veen, Anton Prinssen, Karin Hoppenbrouwers, Britt Hulsen, Mirjam Evers, Jantine van Holten, Femke van der Horst, Lettie van den Berg and Daphne Sjauw. We thank L.M.A. Heunks for critically reviewing the article.

Collaborators in Dutch HFNO COVID-19 study group: Shailin Gajadin: Department of Respiratory Medicine, IJsselland Ziekenhuis, Capelle aan den Ijssel, The Netherlands. Laura Cox: Department of Intensive Care, Admiraal de Ruyter Ziekenhuis, Goes, The Netherlands. Sanjeev Grewal: Department of Intensive Care, Haga Ziekenhuis, Den Haag, The Netherlands. Julien van Oosten: Department of Intensive Care, Erasmus MC, The Netherlands. Imro N. Vlasveld: Department of Internal Medicine, Martini Ziekenhuis, Groningen, The Netherlands. Wouter Jacobs: Department of Respiratory Medicine, Martini Ziekenhuis, Groningen, The Netherlands.

## Supplementary Material

## References

[1] Docherty AB Mulholland RH Lone NI; ISARIC4C Investigators: Changes in in-hospital mortality in the first wave of COVID-19: A multicentre prospective observational cohort study using the WHO Clinical Characterisation Protocol UK. Lancet Respir Med. 2021; 9:773–785 34000238 10.1016/S2213-2600(21)00175-2 PMC8121531

[2] Myers LC Parodi SM Escobar GJ: Characteristics of Hospitalized Adults With COVID-19 in an Integrated Health Care System in California. JAMA. 2020; 323:2195–2198 32329797 10.1001/jama.2020.7202 PMC7182961

[3] Richardson S Hirsch JS Narasimhan M; the Northwell COVID-19 Research Consortium: Presenting Characteristics, Comorbidities, and Outcomes Among 5700 Patients Hospitalized With COVID-19 in the New York City Area. JAMA. 2020; 323:2052–2059 32320003 10.1001/jama.2020.6775 PMC7177629

[4] Zhou F Yu T Du R: Clinical course and risk factors for mortality of adult inpatients with COVID-19 in Wuhan, China: A retrospective cohort study. Lancet. 2020; 395:1054–1062 32171076 10.1016/S0140-6736(20)30566-3 PMC7270627

[5] Mauri T Turrini C Eronia N: Physiologic Effects of High-Flow Nasal Cannula in Acute Hypoxemic Respiratory Failure. Am J Respir Crit Care Med. 2017; 195:1207–1215 27997805 10.1164/rccm.201605-0916OC

[6] Rochwerg B Einav S Chaudhuri D: The role for high flow nasal cannula as a respiratory support strategy in adults: A clinical practice guideline. Intensive Care Med. 2020; 46:2226–2237 33201321 10.1007/s00134-020-06312-y PMC7670292

[7] Clinical management of severe acute respiratory infection (SARI) when COVID-19 disease is suspected: World Health Organization. Available at: https://www.who.int/docs/default-source/coronaviruse/clinical-management-of-novel-cov.pdf. Accessed August 24, 2022

[8] James DC Megan LC Pieter CG.: Management of hospitalised adults with coronavirus disease 2019 (COVID-19): A European Respiratory Society living guideline. Eur Respir J. 2021; 57:2100048. 33692120 10.1183/13993003.00048-2021 PMC7947358

[9] Alhazzani W Moller MH Arabi YM: Surviving sepsis campaign: Guidelines on the management of critically ill adults with coronavirus disease 2019 (COVID-19). Intensive Care Med. 2020; 46:854–887 32222812 10.1007/s00134-020-06022-5 PMC7101866

[10] Gorman E Connolly B Couper K: Non-invasive respiratory support strategies in COVID-19. Lancet Respir Med. 2021; 9:553–556 33872588 10.1016/S2213-2600(21)00168-5 PMC8051928

[11] Network C-IGobotR, the C-ICUI: Clinical characteristics and day-90 outcomes of 4244 critically ill adults with COVID-19: A prospective cohort study. Intensive Care Med. 2021; 47:60–73 33211135 10.1007/s00134-020-06294-x PMC7674575

[12] Weerakkody S Arina P Glenister J: Non-invasive respiratory support in the management of acute COVID-19 pneumonia: Considerations for clinical practice and priorities for research. Lancet Respir Med. 2022; 10:199–213 34767767 10.1016/S2213-2600(21)00414-8 PMC8577844

[13] Bouadma L Mekontso-Dessap A Burdet C; COVIDICUS Study Group: High-dose dexamethasone and oxygen support strategies in intensive care unit patients with severe COVID-19 acute hypoxemic respiratory failure: The COVIDICUS Randomized Clinical Trial. JAMA Intern Med. 2022; 182:906–916 35788622 10.1001/jamainternmed.2022.2168 PMC9449796

[14] Crimi C Noto A Madotto F: High-flow nasal oxygen versus conventional oxygen therapy in patients with COVID-19 pneumonia and mild hypoxaemia: A randomised controlled trial. Thorax. 2022; 78:354–361 35580898 10.1136/thoraxjnl-2022-218806

[15] Frat JP Quenot JP Badie J; SOHO-COVID Study Group and the REVA Network: Effect of high-flow nasal cannula oxygen vs standard oxygen therapy on mortality in patients with respiratory failure due to COVID-19: The SOHO-COVID Randomized Clinical Trial. JAMA. 2022; 328:1212–1222 36166027 10.1001/jama.2022.15613 PMC9516287

[16] Ospina-Tascon GA Calderon-Tapia LE Garcia AF; HiFLo-Covid Investigators: Effect of high-flow oxygen therapy vs conventional oxygen therapy on invasive mechanical ventilation and clinical recovery in patients with severe COVID-19: A randomized clinical trial. JAMA. 2021; 326:2161–2171 34874419 10.1001/jama.2021.20714 PMC8652598

[17] Calligaro GL Lalla U Audley G: The utility of high-flow nasal oxygen for severe COVID-19 pneumonia in a resource-constrained setting: A multi-centre prospective observational study. EClinicalMedicine. 2020; 28:100570 33043285 10.1016/j.eclinm.2020.100570 PMC7536126

[18] Colombo SM Scaravilli V Cortegiani A: Use of high flow nasal cannula in patients with acute respiratory failure in general wards under intensivists supervision: A single center observational study. Respir Res. 2022; 23:171 35754021 10.1186/s12931-022-02090-x PMC9233759

[19] Guy T Creac’hcadec A Ricordel C: High-flow nasal oxygen: A safe, efficient treatment for COVID-19 patients not in an ICU. Eur Respir J. 2020; 56:2001154 32859678 10.1183/13993003.01154-2020 PMC7453734

[20] Hofer MSM Rezek S Ott N: Experience with high-flow nasal oxygen therapy in COVID-19 patients on a regular internal medicine ward. Eur Respir J. 2021; 58:PA1760

[21] ESICM LIVES: 2022: Part 1. Intensive Care Med Exp. 2022; 10:39 36258101 10.1186/s40635-022-00468-1 PMC9579221

[22] Franco C Facciolongo N Tonelli R: Feasibility and clinical impact of out-of-ICU noninvasive respiratory support in patients with COVID-19-related pneumonia. Eur Respir J. 2020; 56:2002130 32747398 10.1183/13993003.02130-2020 PMC7397952

[23] Messika J Ben Ahmed K Gaudry S: Use of high-flow nasal cannula oxygen therapy in subjects with ARDS: A 1-year observational study. Respir Care. 2015; 60:162–169 25371400 10.4187/respcare.03423

[24] World Medical Association: World Medical Association Declaration of Helsinki: Ethical principles for medical research involving human subjects. JAMA. 2013; 310:2191–2194 24141714 10.1001/jama.2013.281053

[25] von Elm E Altman DG Egger M; STROBE Initiative: Strengthening the Reporting of Observational Studies in Epidemiology (STROBE) statement: Guidelines for reporting observational studies. BMJ. 2007; 335:806–808 17947786 10.1136/bmj.39335.541782.AD PMC2034723

[26] Vincent JL de Mendonca A Cantraine F: Use of the SOFA score to assess the incidence of organ dysfunction/failure in intensive care units: Results of a multicenter, prospective study. Working group on “sepsis-related problems” of the European Society of Intensive Care Medicine. Crit Care Med. 1998; 26:1793–1800 9824069 10.1097/00003246-199811000-00016

[27] Knight SR Ho A Pius R; ISARIC4C investigators: Risk stratification of patients admitted to hospital with covid-19 using the ISARIC WHO Clinical Characterisation Protocol: Development and validation of the 4C Mortality Score. BMJ. 2020; 370:m3339 32907855 10.1136/bmj.m3339 PMC7116472

[28] Gupta RK Harrison EM Ho A; ISARIC4C Investigators: Development and validation of the ISARIC 4C Deterioration model for adults hospitalised with COVID-19: A prospective cohort study. Lancet Respir Med. 2021; 9:349–359 33444539 10.1016/S2213-2600(20)30559-2 PMC7832571

[29] Roca O Caralt B Messika J: An index combining respiratory rate and oxygenation to predict outcome of nasal high-flow therapy. Am J Respir Crit Care Med. 2019; 199:1368–1376 30576221 10.1164/rccm.201803-0589OC

[30] Yehya N Harhay MO Curley MAQ: Reappraisal of ventilator-free days in critical care research. Am J Respir Crit Care Med. 2019; 200:828–836 31034248 10.1164/rccm.201810-2050CP PMC6812447

[31] Demoule A Vieillard Baron A Darmon M: High-flow nasal cannula in critically ill patients with severe COVID-19. Am J Respir Crit Care Med. 2020; 202:1039–1042 32758000 10.1164/rccm.202005-2007LE PMC7528777

[32] Grieco DL Menga LS Cesarano M; COVID-ICU Gemelli Study Group: Effect of Helmet noninvasive ventilation vs high-flow nasal oxygen on days free of respiratory support in patients with COVID-19 and moderate to severe hypoxemic respiratory failure: The HENIVOT Randomized Clinical Trial. JAMA. 2021; 325:1731–1743 33764378 10.1001/jama.2021.4682 PMC7995134

[33] Perkins GD Ji C Connolly BA; RECOVERY-RS Collaborators: Effect of noninvasive respiratory strategies on intubation or mortality among patients with acute hypoxemic respiratory failure and COVID-19: The RECOVERY-RS Randomized Clinical Trial. JAMA. 2022; 327:546–558 35072713 10.1001/jama.2022.0028 PMC8787685

[34] Jackson JA Spilman SK Kingery LK: Implementation of High-Flow Nasal Cannula Therapy Outside the Intensive Care Setting. Respir Care. 2021; 66:357–365 32843505 10.4187/respcare.07960

[35] Issa I Soderberg M: High-flow nasal oxygen (HFNO) for patients with COVID-19 outside intensive care units. Respir Med. 2021; 187:106554 34340173 10.1016/j.rmed.2021.106554 PMC8317480

[36] Sykes DL Crooks MG Thu Thu K: Outcomes and characteristics of COVID-19 patients treated with continuous positive airway pressure/high-flow nasal oxygen outside the intensive care setting. ERJ Open Res. 2021; 7:00318–02021 10.1183/23120541.00318-2021 PMC8287575 34611525

[37] Kang BJ Koh Y Lim CM: Failure of high-flow nasal cannula therapy may delay intubation and increase mortality. Intensive Care Med. 2015; 41:623–632 25691263 10.1007/s00134-015-3693-5

[38] Oczkowski S Ergan B Bos L: ERS clinical practice guidelines: High-flow nasal cannula in acute respiratory failure. Eur Respir J. 2022; 59:2101574 34649974 10.1183/13993003.01574-2021

[39] Investigators AC-CCOS: African COVID-critical care outcomes study investigator. Patient care and clinical outcomes for patients with COVID-19 infection admitted to African high-care or intensive care units (ACCCOS): A multicentre, prospective, observational cohort study. Lancet. 2021; 397:1885–1894 34022988 10.1016/S0140-6736(21)00441-4 PMC8137309

[40] Renda T Scala R Corrado A; Scientific Group on Respiratory Intensive Care of the Italian Thoracic Society (ITS-AIPO): Adult pulmonary intensive and intermediate care units: The Italian Thoracic Society (ITS-AIPO) position paper. Respiration. 2021; 100:1027–1037 34102641 10.1159/000516332

[41] Mellado-Artigas R Ferreyro BL Angriman F; COVID-19 Spanish ICU Network: High-flow nasal oxygen in patients with COVID-19-associated acute respiratory failure. Crit Care. 2021; 25:58 33573680 10.1186/s13054-021-03469-w PMC7876530

[42] de Lange DW Soares M Pilcher D: ICU beds: Less is more? No. Intensive Care Med. 2020; 46:1597–1599 32458052 10.1007/s00134-020-06089-0 PMC7248458

[43] Arabi YM Derde LPG Timsit JF: How COVID-19 will change the management of other respiratory viral infections. Intensive Care Med. 2021; 47:1148–1151 34379151 10.1007/s00134-021-06491-2 PMC8355267

[44] Bein T Bienvenu OJ Hopkins RO: Focus on long-term cognitive, psychological and physical impairments after critical illness. Intensive Care Med. 2019; 45:1466–1468 31384964 10.1007/s00134-019-05718-7

[45] Heesakkers H van der Hoeven JG Corsten S: Mental health symptoms in family members of COVID-19 ICU survivors 3 and 12 months after ICU admission: A multicentre prospective cohort study. Intensive Care Med. 2022; 48:322–331 35103824 10.1007/s00134-021-06615-8 PMC8804080

[46] Brochard L Slutsky A Pesenti A: Mechanical ventilation to minimize progression of lung injury in acute respiratory failure. Am J Respir Crit Care Med. 2017; 195:438–442 27626833 10.1164/rccm.201605-1081CP

[47] Marini JJ Gattinoni L: Management of COVID-19 respiratory distress. JAMA. 2020; 323:2329–2330 32329799 10.1001/jama.2020.6825

[48] Tobin MJ Laghi F Jubran A: Caution about early intubation and mechanical ventilation in COVID-19. Ann Intensive Care. 2020; 10:78 32519064 10.1186/s13613-020-00692-6 PMC7281696

[49] Prakash J Bhattacharya PK Yadav AK: ROX index as a good predictor of high flow nasal cannula failure in COVID-19 patients with acute hypoxemic respiratory failure: A systematic review and meta-analysis. J Crit Care. 2021; 66:102–108 34507079 10.1016/j.jcrc.2021.08.012 PMC8424061

[50] Haukoos JS Lewis RJ: The propensity score. JAMA. 2015; 314:1637–1638 26501539 10.1001/jama.2015.13480 PMC4866501

[51] Liu L Xie J Wu W: A simple nomogram for predicting failure of non-invasive respiratory strategies in adults with COVID-19: A retrospective multicentre study. Lancet Digit Health. 2021; 3:e166–e174 33573999 10.1016/S2589-7500(20)30316-2 PMC7906717

[52] Turtle L Thorpe M Drake TM; ISARIC4C investigators: Outcome of COVID-19 in hospitalised immunocompromised patients: An analysis of the WHO ISARIC CCP-UK prospective cohort study. PLoS Med. 2023; 20:e1004086 36719907 10.1371/journal.pmed.1004086 PMC9928075

[53] Wang B Yu Y Yu Y: Clinical features and outcomes of hospitalized patients with COVID-19 during the omicron wave in Shanghai, China. J Infect. 2023; 86:e27–e29 36257856 10.1016/j.jinf.2022.08.001 PMC9550297

