# Supplementary material for: Safety and Outcome of High-Flow Nasal Oxygen Therapy Outside ICU Setting in Hypoxemic Patients With COVID-19*
Source: Crit Care Med. 2023 Oct 19;52(1):31–43. doi: 10.1097/CCM.0000000000006068 (PMC10715700; doi:10.1097/CCM.0000000000006068)
Supplement: Supplementary file 1 [file ccm-52-31-s001.pdf]

Online Supplement

Table of Contents

STROBE Statement 2

Figure S1 5

Propensity matching method 6

Table S1 6

Table S2 7

Table S3 9

Table S4 11

Table S5 13

Table S6 16

Table S7 19

Table S8 21

Table S9 24

Table S10 27

Table S11 29

References 30

STROBE Statement—Checklist of items that should be included in reports of ***cohort studies***

	Item No	Recommendation	Page No
**Title and abstract**	1	(*a*) Indicate the study's design with a commonly used term in the title or the abstract	4
		(*b*) Provide in the abstract an informative and balanced summary of what was done and what was found	4
**Introduction**			
Background/rationale	2	Explain the scientific background and rationale for the investigation being reported	6
Objectives	3	State specific objectives, including any prespecified hypotheses	6-7
**Methods**			
Study design	4	Present key elements of study design early in the paper	7-8
Setting	5	Describe the setting, locations, and relevant dates, including periods of recruitment, exposure, follow-up, and data collection	7-8
Participants	6	(*a*) Give the eligibility criteria, and the sources and methods of selection of participants. Describe methods of follow-up	7-8
		(*b*) For matched studies, give matching criteria and number of exposed and unexposed	9 and supplements
Variables	7	Clearly define all outcomes, exposures, predictors, potential confounders, and effect modifiers. Give diagnostic criteria, if applicable	7, table 1, supplements
Data sources/ measurement	8*	For each variable of interest, give sources of data and details of methods of assessment (measurement). Describe comparability of assessment methods if there is more than one group	7,8 and data dictionary
Bias	9	Describe any efforts to address potential sources of bias	7-9

Study size	10	Explain how the study size was arrived at	9
Quantitative variables	11	Explain how quantitative variables were handled in the analyses. If applicable, describe which groupings were chosen and why	8,9
Statistical methods	12	(*a*) Describe all statistical methods, including those used to control for confounding	8,9
		(*b*) Describe any methods used to examine subgroups and interactions	8,9
		(*c*) Explain how missing data were addressed	9
		(*d*) If applicable, explain how loss to follow-up was addressed	NA
		(*e*) Describe any sensitivity analyses	8,9
**Results**			
Participants	13*	(a) Report numbers of individuals at each stage of study—eg numbers potentially eligible, examined for eligibility, confirmed eligible, included in the study, completing follow-up, and analysed	10, Fig 1.
		(b) Give reasons for non-participation at each stage	NA
		(c) Consider use of a flow diagram	Fig.1
Descriptive data	14*	(a) Give characteristics of study participants (eg demographic, clinical, social) and information on exposures and potential confounders	10-12, table 2-4, and supplements
		(b) Indicate number of participants with missing data for each variable of interest	Table S3
		(c) Summarise follow-up time (eg, average and total amount)	10
Outcome data	15*	Report numbers of outcome events or summary measures over time	10-12, table 2-4, and supplements

Main results	16	(*a*) Give unadjusted estimates and, if applicable, confounder-adjusted estimates and their precision (eg, 95% confidence interval). Make clear which confounders were adjusted for and why they were included	10-12, table 2-4, and supplements
		(*b*) Report category boundaries when continuous variables were categorized	NA
		(*c*) If relevant, consider translating estimates of relative risk into absolute risk for a meaningful time period	NA
Other analyses	17	Report other analyses done—eg analyses of subgroups and interactions, and sensitivity analyses	10-12, table 2-4, and supplements
**Discussion**			
Key results	18	Summarise key results with reference to study objectives	14
Limitations	19	Discuss limitations of the study, taking into account sources of potential bias or imprecision. Discuss both direction and magnitude of any potential bias	16-17
Interpretation	20	Give a cautious overall interpretation of results considering objectives, limitations, multiplicity of analyses, results from similar studies, and other relevant evidence	14-17
Generalisability	21	Discuss the generalisability (external validity) of the study results	14-17
**Other information**			
Funding	22	Give the source of funding and the role of the funders for the present study and, if applicable, for the original study on which the present article is based	2

*Give information separately for exposed and unexposed groups. NA: Not Applicable.

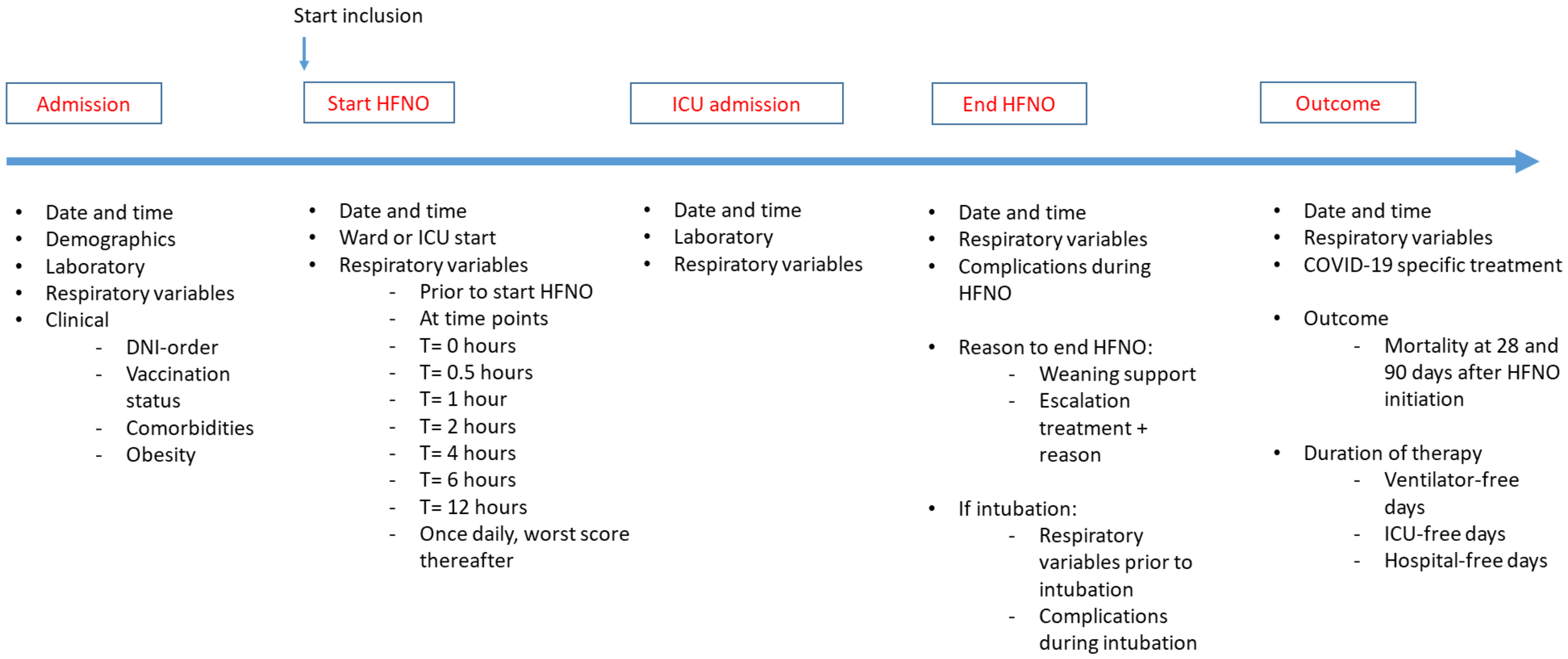

Figure S1: Overview of data collection during study period. Respiratory variables include: FiO_2, SpO_2, SpO_2/FiO_2 ratio, respiratory rate, ROX-index, flow and type of support. Obesity was defined as Body Mass Index (BMI) ≥30.

Propensity matching method

The propensity score to start HFNO therapy in ICU was calculated for each patient, based on relevant confounders with a clear association to outcome in COVID-19 patients (listed in table M1 below).

Table S1. **Variables used for propensity matching.**
Age (1)
Sex (2, 3)
Body Mass Index (BMI) (4, 5)
Number of comorbidities (from Charlson comorbidity index)(1, 6)
CRP at HFNO start (1, 6)
Urea at HFNO start (1, 6)
Leukocyte count at HFNO start (7, 8)
Lymphocyte count at HFNO start (1, 6)
Thrombocytopenia at HFNO start (9)
Duration of illness at HFNO start (days) (10, 11)
Respiratory rate prior to start HFNO (1, 9)
FiO_2 prior to start HFNO (2, 12, 13)
SpO_2 prior to start HFNO (12)

Table S2. **Overview of local HFNO practice outside ICU during the study period.**

Centre	Patients without DNI order	Ward starters	HFNO start on ward in patients without DNI order	HFNO start on ICU, continued on ward	Flow limit on <60 L/min	Flow start HFNO L/min	Flow at phasing out HFNO L/min	Cont. monitoring respiratory rate and SpO_2	Cont. monitoring blood-pressure or telemetry	Standard frequency of vital parameter control in HFNO patients	Use of the ROX index	Standard ICU consul-tation at HFNO start
**1**	249 (41.0%)	229 (60.4%)	Yes	Yes	Yes (40L)	40	40	Yes	No	First 24 hours HFNO: at 0.5, 1, 2, 4, 6, 12 and 24 hours. Then 3 times daily.	Yes	No
**2**	53 (8.7%)	43 (11.3%)	Yes	Yes	No	60	30	Yes	Yes	First 4 hours: every hour, then 3 times daily.	Yes	No
**3**	47 (7.7%)	7 (1.8%)*	No	Yes	No	50	NA	Yes	No	First 24 hours: every 2 hours, then 3 times daily	No	NA
**4**	11 (1.8%)	11 (2.9%)	Yes	Yes	No	40	<40	Yes	Yes	4 times daily	No	No
**5**	51 (8.4%)	0 (0%)	No	No	NA	NA	NA	NA	NA	NA	No	NA
**6**	36 (5.9%)	3 (0.8%)*	No	Yes	No	60	40	No	No	3 times daily	No	NA
**7**	61 (10.0%)	0 (0%)	No	No	NA	NA	NA	NA	NA	NA	Yes	NA
**8**	13 (2.1%)	7 (1.8%)	Yes	Yes	No	40	40	No	No	First 24 hours HFNO: at 0.5, 1, 2, 4, 6, 12 and 24 hours. Then 4 times daily.	No	No
**9**	82 (13.5%)	79 (20.8%)	Yes	Yes	No	40	40	No	No	6 times daily	No	No
**10**	5 (0.8%)	0 (0%)	No	No	NA	NA	NA	NA	NA	NA	No	NA
**Total**	**608**	**379**	**5/10**	**7/10**	**1/7**	**-**	**-**	**4/7**	**2/7**	**-**	**3/7**	**0/5**

Categorical variables are presented as number with percentage. Abbreviations: DNI: Do not intubate order, HFNO: High-flow nasal oxygen, ICU: Intensive Care unit, L/min: litre per minute, RR: respiratory rate, SpO_2: oxygen saturation. *Some patients that started HFNO on the ward, initially had a DNI-order which was lifted later during admission.

Table S3. **Overview of staffing on Pulmonary ward/ Intermediate care unit and Intensive Care Units during study period.**

	Pulmonary Intermediate Care Unit nested in regular pulmonary ward					Intensive Care Unit					
**Centre**	**Mean capacity (HFNO beds)**	**Day and night-time consultants on call**	**Day and night-time residents (night-time on call)**	**Daytime nurse-patient ratio**	**Night-time nurse-patient ratio**	**Mean capacity**	**Day and night-time intensivists**	**Day and night-time residents**	**Day time nurse-patient ratio**	**Night-time nurse-patient ratio**	**Non-ICU supportive staff per shift***
**1**	34 (12)	1 - 1	3 – 1	1:4	1:4.5	20	3 – 1.5	2 - 1	1: 2.2	1: 2.2	3
**2**	32 (6)	2 – 2	3 – 2	1:3.5	1:4.5	56	4 – 1	5 - 3	1: 1.8	1: 2.9	4
**3**	36 (3)	1 - 1	3 – 1	1:3.6	1:6	20	2 – 1	3 – 2	1: 2.2	1: 2.9	4
**4**	20 (10)	2 - 2	0 – 0	1:3	1.6.6	12	2 – 1	1 – 1	1: 1.3	1: 2.0	2
**5**	NA	NA	NA	NA	NA	20	2 – 1	2 – 1	1: 2.5	1: 3.3	5
**6**	20 (2)	1 - 1	2 – 1	1:3.3	1:6.6	13	2 – 1	3 – 1	1: 1.6	1: 2.6	3
**7**	NA	NA	NA	NA	NA	20	2 – 1	2 – 1	1: 2.5	1: 3.3	2
**8**	12 (8)	1 - 1	1 - 1	1:3	1:6	8	2 – 1	1 – 0	1: 1.3	1: 2.0	2
**9**	28 (10)	2 - 1	3 - 1	1:3	1:4.6	16	3 – 1	3 – 2	1: 1.8	1: 2.3	7
**10**	NA	NA	NA	NA	NA	18	2 – 1	3 – 2	1: 1.6	1: 1.8	7

Abbreviations: HFNO: High-flow nasal oxygen, ICU: Intensive Care unit. NA: Not Applicable. In the Netherlands, there are no respiratory therapists in hospital. *Non-ICU staff included anaesthesia nurses employed for non-ICU nurse tasks during COVID-19 peaks.

Table S4. **Detailed overview of standard local medical treatment of patients with COVID-19 treated with HFNO during study period.**

Centre	Dexame-thasone 6mg/day, 10 days	Patients receiving dexame-thasone	IL-6 blocking agents[×], with indications	Patients receiving IL-6 antagonist	Methylpred-nisolone 1000 mg /day, 3 days	LMWH low dose i.e. dalteparin 1d5000 IU	LMWH in-termediate dose i.e. dalteparin 2d 5000 IU	LMWH in therapeutic dose (standard)	Azytromycin, (hydroxy) chloroquin, or any other*	Antibiotics	Awake proning
1	Yes	248/249 (99.6%)	Yes, if oxygen ≥6L/min	149/249[±] (59.8%)	No	Yes	Yes, ICU only	No	No	No	No
2	Yes	52/53 (98.1%)	Yes, if CRP ≥75 and oxygen ≥6L/min	46/53 (86.8%)	No	Yes	Yes, ICU only	No	No	No	No
3	Yes	43/47 (91.5%)	Yes, if oxygen ≥6L/min	30/47 (63.8%)	No	No	Yes	No	No	No	No
4	Yes	11/11 (100%)	Yes, if CRP ≥75 and any hypoxemia	4/11 (36.4%)	No	No	Yes	No	No	Yes [+]	No
5	Yes	51/51 (100%)	Yes, if oxygen ≥6L/min	41/51 (80.4%)	No	No	Yes, ICU only	No	No	No	No
6	Yes	36/36 (100%)	Yes, at ICU admission	23/36[±] (63.9%)	No	Yes	Yes, ICU only	No	No	No	Yes, ICU only
7	Yes	61/61 (100%)	Yes	61/61 (100%)	No	Yes	No	Yes, ICU only	No	Yes [+]	No
8	Yes	12/13 (92.9%)	Yes, if CRP ≥75 and oxygen ≥6L/min	9/13 (69.2%)	No	No	Yes	No	No	Yes[‡]	No
9	Yes	82/82 (100%)	Yes, if CRP ≥75	22/82 (26.8%)	No	Yes	Yes	No	No	No	No
10	Yes	5/5 (100%)	Yes, if CRP ≥75 and oxygen ≥6L/min	4/5 (80%)	No	No	Yes	No	No	No	No
**Total**	**10/10**	**601/608 (98.8%)**	**10/10**	**389/608 (64.0%)**	**0/10**	**5/10**	**9/10**	**1/10**	**0/10**	**3/10**	**1/10**

[×]: Tocilizumab 600 mg or sarilumab 400 mg once; was implemented during the study. *: All hospitals used remdesivir until proven ineffective. ±: Unknown in N=3 patients. +: Ceftriaxone and Ciprofloxacin. ‡ Ceftriaxone. Abbreviations: CRP: C-reactive protein. IU: International Units. LMWH: Low-Molecular Weight Heparin. IL-6: interleukin-6.

Table S5. **Characteristics of the study cohort**

	Overall (n=608)	Ward-starters (n=379)	ICU-starters (n=229)	P-value	Missing
Age	61 (53 - 68)	61 (53 - 68)	61 (52 – 67)	0.61	0
Male sex	417 (68.6)	247 (65.2)	170 (74.2)	0.03	0
Obesity	252 (41.4)	146 (38.5)	106 (46.3)	0.67	53 (8.7)
Number of comorbidities				0.63	1 (0.2)
0	319 (52.5)	213 (56.2)	106 (46.3)		
1	178 (29.3)	105 (27.7)	73 (31.9)		
≥2	110 (18.1)	61 (16.1)	49 (21.4)		
SOFA score at hospital admission	3 (2 – 3)	2 (2 - 3)	3 (3 - 3)	<0.001	36 (5.9)
Non-respiratory SOFA score at hospital admission	0 (0 - 1)	0 (0 - 1)	0 (0 - 1)	0.12	36 (5.9)
4C mortality score at hospital admission	10 (7 - 12)	9 (7 - 12)	10 (8 – 12)	0.02	66 (10.9)
4C deterioration score at hospital admission	0.55 (0.43 - 0.65)	0.52 (0.41 - 0.63)	0.59 (0.48 - 0.69)	<0.001	76 (12.5)
Variables at hospital admission					
S/F ratio	232 (123 - 288)	249 (211 - 291)	125 (116 - 238)	<0.001	3 (0.5)
FiO_2	0.41 (0.33 – 0.80)	0.37 (0.33 – 0.45)	0.80 (0.41 – 0.80)	<0.001	3 (0.5)
PaO_2 (mmHg)	65.3 (57.0 - 76.8)	68.0 (59.3 - 77.5)	64.0 (55.0 - 73.6)	0.001	61 (10.0)
P/F ratio	170 (111 - 243)	200 (149 - 262)	110 (82.5 - 173)	<0.001	63 (10.4)
Respiratory rate	25 (22 - 30)	25 (20 - 30)	28 (23 - 32)	<0.001	5 (0.8)
ROX index	8.74 (4.95 - 12.7)	9.76 (7.24 - 13.4)	5.23 (3.83 - 10.6)	<0.001	9 (1.5)
Variables prior to HFNO start					
S/F ratio	123 (116 - 204)	191 (123 – 220)	116 (113 - 120)	<0.001	1 (0.2)
FiO_2	0.80 (0.45 – 0.80)	0.60 (0.41 – 0.80)	0.80 (0.80 - 0.80)	<0.001	1 (0.2)
Respiratory rate (/min)	28 (24 - 32)	26 (24 – 31)	30 (25 - 35)	<0.001	8 (1.3)
ROX index	5.1 (3.9 - 7.5)	6.5 (5.0 - 8.5)	3.9 (3.4 - 4.7)	<0.001	9 (1.5)
Laboratory values at HFNO start					
CRP (µmol/L)	112 (66 - 178)	109 (66 - 169)	116 (66 - 190)	0.296	2 (0.3)
Urea (mmol/L)	6.3 (4.7 - 8.6)	6.00 (4.3 - 8.1)	6.80 (5.2 - 9.7)	<0.001	6 (1.0)
Ferritin (mg/L)	1080 (498 - 2070)	961 (458 - 2200)	1240 (611 - 1930)	0.38	150 (24.7)
Leukocytes (10^9/L)	7.6 (5.5 - 10.2)	7.1 (5.3 - 9.2)	8.4 (6.1 - 11.5)	<0.001	14 (6.5)
Lymphocytes (10^9/L)	0.8 (0.6 - 1.1)	0.8 (0.6 - 1.1)	0.8 (0.6 - 1.2)	0.34	63 (10.4)
Thrombocytes (10^9/L)	227 (180 - 290)	216 (173 - 264)	254 (202 - 314)	<0.001	16 (7.5)

Outcome					
Duration of symptoms at hospital admission (days)	8 (7 - 10)	8 (7 - 10)	9 (7 – 10)	0.24	27 (4.4)
Time between hospital admission and start HFNO (hours)	17.0 (2.0 - 47.0)	18.0 (3.0 - 42.7)	16.0 (2.0 - 59.0)	0.33	4 (0.8)
ICU admission	415 (68.3)	186 (49.1)	229 (100)	<0.001	0
Time admitted until ICU admission (hours)	35 (5 - 79)	47 (24 – 96)	12 (2- 59)	<0.001	23 (3.7)
Intubation	277 (45.6)	139 (36.7)	138 (60.3)	<0.001	0
Intubation ≤4 hours after HFNO start	28 (4.6)	9 (2.4)	19 (8.3)	<0.001	2 (0.7)
Intubation ≤6 hours after HFNO start	43 (7.1)	14 (3.7)	29 (12.7)	0.001	2 (0.7)
Intubation ≤2 hours after ICU admission	43 (7.1)	36 (9.5)	7 (3.1)	0.003	2 (0.7)
Intubation ≤4 hours after ICU admission	74 (12.2)	57 (15.0)	17 (7.5)	0.01	2 (0.7)
Mortality prior to intubation	0	0	0	NA	0
In-hospital mortality	56 (9.2)	22 (5.8)	34 (14.8)	<0.001	0
28-day mortality after HFNO start	38 (6.3)	13 (3.4)	25 (10.9)	<0.001	20 (3.3)
90-day mortality after HFNO start	56 (9.2)	22 (5.8)	34 (14.8)	<0.001	41 (6.7)
Hospital-free days at day 28 after admission (days)	13 (0 – 19)	15 (3 – 19)	6 (0 – 17)	<0.001	3 (0.5)
Hospital-free days at day 60 after admission (days)	45 (26 – 51)	47 (35 – 51)	38 (9 – 49)	<0.001	3 (0.5)
ICU-free days at day 28 after HFNO start (days)	23 (11 – 28)	28 (17 - 28)	18 (0 - 23)	<0.001	3 (0.5)
ICU-free days at day 60 after HFNO start (days)	55 (43 – 60)	60 (49 - 60)	50 (30.5 - 55)	<0.001	3 (0.5)
Ventilator-free days at day 28 after HFNO start (days)	28 (16 – 28)	28 (21 - 28)	22 (7 - 28)	<0.001	1 (0.2)
Ventilator-free days at day 60 after HFNO start (days)	60 (48 – 60)	60 (53 - 60)	54 (36 - 60)	<0.001	1 (0.2)

Categorical variables are presented as number with percentage, continuous variables are presented as median with interquartile range. Differences between groups regarding continuous variables were analysed Mann–Whitney U test. Differences between groups regarding categorical variables were analysed using the Chi-squared test, or with Fisher's exact test in case of a cell with less than 5 subjects. Abbreviations: SOFA: Sequential Organ Failure Assessment, HFNO: High-flow Nasal Oxygen, ICU: Intensive Care Unit, S/F ratio: SpO_2-FiO_2 ratio, P/F ratio: PaO_2-FiO_2 ratio, CRP: C-reactive Protein

Table S6. **Characteristics of the propensity matched cohort, ward- vs. ICU starters**

	Ward-starters (n=107)	ICU- starters (n=107)	P-value
Age	63.0 (54.0 - 70.0)	63.0 (53.0 - 69.0)	0.62
Male sex	70 (65.4)	82 (76.6)	0.09
Obesity	45 (45.9)	45 (42.5)	0.68
Number of comorbidities			0.94
0	53 (49.5)	49 (46.2)	
1	30 (28.0)	31 (29.2)	
≥2	24 (22.4)	26 (24.5)	
SOFA score at hospital admission	3 (3 - 4)	3 (3 – 4)	0.17
Non-respiratory SOFA at hospital admission	0 (0 - 1)	0 (0 - 1)	0.04
4C mortality score at hospital admission	11 (8- 13)	10 (8 - 12)	0.61
4C deterioration score at hospital admission	0.58 (0.45 - 0.67)	0.58 (0.47 - 0.67)	0.88
Variables at hospital admission			
S/F ratio	204 (120 - 283)	160 (119 - 236)	0.35
FiO_2	0.45 (0.33 - 0.80)	0.60 (0.41 - 0.80)	0.36
PaO_2 (mmHg)	73.0 (59.8 - 88.5)	65.0 (56.1 - 77.0)	0.12
P/F ratio	153 (112 - 226)	123 (89 - 200)	0.08
Respiratory rate (/min)	26.5 (22 - 32)	26 (22 - 30)	0.44
ROX index	7.1 (4.6 – 12.7)	6.0 (4.1 – 11.8)	0.95
Variables prior to HFNO start			
S/F ratio	119 (115 - 121)	118 (114 – 121)	0.93
FiO_2	0.80 (0.80 - 0.80)	0.80 (0.80 - 0.80)	0.97
Respiratory rate (/min)	28 (24 - 32)	28 (24 - 32)	0.88
ROX index	4.3 (3.7 - 5.1)	4.2 (3.8 – 5.0)	0.70
Laboratory at HFNO start			
CRP (µmol/L)	112 (49.8 - 188)	98.0 (54.5 - 168)	0.66
Urea (mmol/L)	6.8 (4.9 - 8.2)	7.4 (5.9 - 9.7)	<0.01
Ferritin (mg/L)	1013 (414 - 2190)	1411 (645 - 2020)	0.77
Leukocytes (10^9/L)	7.1 (5.7 - 9.2)	8.0 (5.6 - 10.8)	0.06
Lymphocytes (10^9/L)	0.7 (0.6 - 0.9)	0.8 (0.6 - 1.2)	0.24
Thrombocytes (10^9/L)	220 (174 - 290)	226 (187 - 286)	0.95

Outcome			
Duration of symptoms at hospital admission (days)	8.0 (6.3 - 10.0)	9.0 (7.0 - 10.0)	0.25
Time between hospital admission and start HFNO (hours)	5.0 (2.0 – 59.0)	16.0 (2.0 - 67.0)	0.69
ICU admission	68 (63.6)	107 (100)	NA
Time on HFNO until ICU admission (hours)	16.0 (4.0 - 43.0)	-	NA
Intubation	57 (53.3)	64 (59.8)	0.42
Intubation ≤4 hours after HFNO start	7 (6.5)	7 (6.5)	1
Intubation ≤6 hours after HFNO start	11 (10.3)	13 (12.1)	0.83
Intubation ≤2 hours after ICU admission	16 (15.0)	1 (0.9)	0.001
Intubation ≤4 hours after ICU admission	25 (23.4)	7 (6.5)	<0.01
Time on HFNO until intubation (hours)	37.2 (9.3 - 85.5)	23.7 (9.0 - 52.6)	0.17
Time between ICU admission and intubation (hours)	5.3 (1.5 - 23.0)	23.5 (9.8 - 51.3)	0.01
Mortality prior to intubation	0	0	NA
Prone positioning after intubation	47 (43.9)	51 (47.6)	0.50
In hospital mortality	14 (13.1)	19 (17.8)	0.45
28-day mortality after HFNO start	8 (7.5)	13 (13.0)	0.24
90-day mortality after HFNO start	13 (12.6)	19 (19.8)	0.33
Hospital-free days at day 28 after admission (days)	9 (0 - 16)	4 (0 - 17)	0.62
Hospital-free days at day 60 after admission (days)	41 (19 - 48)	36 (0 - 49)	0.29
ICU-free days at day 28 after HFNO start (days)	21 (10 - 28)	17 (0 - 24)	<0.001
ICU-free days at day 60 after HFNO start (days)	53 (42 - 60)	49 (5.5 - 56)	0.02
Ventilator-free days at day 28 after HFNO start (days)	24 (13 - 28)	22 (1.5 - 28)	0.13
Ventilator-free days at day 60 after HFNO start (days)	56 (45 - 60)	54 (13.5 - 60)	0.13

Categorical variables are presented as number with percentage, continuous variables are presented as median with Interquartile range. Differences between groups regarding continuous variables were analysed by Wilcoxon signed rank. Differences between groups regarding categorical variables were analysed using a McNemar test. Abbreviations: SOFA: Sequential Organ Failure Assessment, HFNO: High-flow Nasal Oxygen, ICU: Intensive Care Unit, S/F ratio: SpO_2-FiO_2 ratio, P/F ratio: PaO_2-FiO_2 ratio, CRP: C-reactive Protein

Table S7. **Distribution of centers within the matched cohort, compared between ward- and ICU-starters.**

Institute	ward (n=107)	ICU (n=107)
1	44 (41.1)	10 (9.3)
2	37 (34.6)	5 (4.7)
3	4 (3.7)	19 (17.8)
4	3 (2.8)	0 (0.0)
5	0 (0.0)	20 (18.7)
6	3 (2.8)	16 (15.0)
7	0 (0.0)	32 (29.9)
8	4 (3.7)	1 (0.9)
9	12 (1.2)	2 (1.9)
10	0 (0.0)	2 (1.9)

Numbers of patients with percentages between brackets.

Table S8. **Characteristics of ICU-starters, non-matched vs. propensity matched**

	Non-matched (n=122)	Matched (n=107)	P-value
Age	60 (52 - 65)	63 (53 - 69)	0.14
Male sex	88 (72.1)	82 (76.6)	0.53
Obesity	61 (50.4)	45 (42.5)	0.29
Number of comorbidities			0.52
0	57 (46.7)	49 (46.2)	
1	42 (34.4)	31 (29.2)	
≥2	23 (18.9)	26 (24.5)	
SOFA score at hospital admission	3 (2 – 3)	3 (3 – 3.8)	0.37
Non-respiratory SOFA score at hospital admission	0 (0 - 1)	0 (0 - 1)	0.14
4C mortality score at hospital admission	10 (8 - 12)	10 (8 – 12)	0.35
4C deterioration score at hospital admission	0.60 (0.49 – 0.72)	0.58 (0.47 – 0.67)	0.11
Variables at hospital admission			
S/F ratio	121 (116 - 239)	160 (119 - 236)	0.04
FiO_2	0.80 (0.40 - 0.80)	0.60 (0.41 - 0.80)	0.12
PaO_2 (mmHg)	62 (55 - 71)	65 (56 - 77)	0.08
P/F ratio	101 (78 - 167)	123 (89 - 200)	0.02
Respiratory rate (/min)	30 (24 - 34)	26 (22 - 30)	0.02
ROX index	4.7 (3.6 - 10.2)	6.0 (4.1 - 11.5)	0.02
Variables prior to HFNO start			
S/F ratio	115 (111 - 119)	118 (114 - 121)	0.001
FiO_2	0.80 (0.80 - 0.80)	0.80 (0.80 - 0.80)	<0.001
Respiratory rate (/min)	31 (26 - 38)	28 (24 - 32)	<0.001
ROX index	3.7 (3.1 – 4.2)	4.2 (3.8 – 5.0)	<0.001
Outcome			
Duration of symptoms at hospital admission (days)	8 (7 - 11)	9 (7 - 10)	0.70
Time between hospital admission and start HFNO (hours)	10.0 (1.0 – 51.8)	16.0 (2.0 – 67.0)	0.26
Intubation	74 (60.7)	64 (59.8)	0.99
Intubation ≤4 hours after HFNO start	12 (9.8)	7 (6.5)	0.51
Intubation ≤6 hours after HFNO start	16 (13.1)	13 (12.1)	0.98
Intubation ≤2 hours after ICU admission	6 (5.0)	1 (0.9)	0.17

Intubation ≤4 hours after ICU admission	10 (8.3)	7 (6.6)	0.83
In-hospital mortality	16 (13.1)	19 (17.8)	0.43
28-day mortality after HFNO start	12 (9.8)	13 (13.0)	0.75
90-day mortality after HFNO start	15 (12.3)	19 (17.8)	0.41
Hospital-free days at day 28 after admission (days)	7 (0 - 16)	4 (0 - 17)	0.61
Hospital-free days at day 60 after admission (days)	39 (19 - 48)	36 (0 - 49)	0.55
ICU-free days at day 28 after HFNO start (days)	19 (2.8 - 23)	17 (0 - 24)	0.61
ICU-free days at day 60 after HFNO start (days)	51 (34.5)	49 (5.5 - 56)	0.49
Ventilator-free days at day 28 after HFNO start (days)	23 (10.8 - 28)	22 (1.5 – 28)	0.30
Ventilator-free days at day 60 after HFNO start (days)	55 (42 - 60)	54 (13.5 - 60)	0.26

Categorical variables are presented as number with percentage, continuous variables are presented as median with Interquartile range. Differences between groups regarding continuous variables were analysed with the Mann–Whitney U test. Differences between groups regarding categorical variables were analysed using the Chi-squared test, or with Fisher's exact test in case of a cell with less than 5 subjects. Abbreviations: SOFA: Sequential Organ Failure Assessment, HFNO: High-flow Nasal Oxygen, ICU: Intensive Care Unit, S/F ratio: SpO_2-FiO_2 ratio, CRP: C-reactive Protein

Table S9. **Characteristics of ward-starters, non-matched vs. propensity matched**

	Non-matched (n=272)	Matched (n=107)	P-value
Age	60 (53 - 68)	63 (54 - 70)	0.19
Male sex	177 (65.1)	70 (65.4)	0.99
Obesity	101 (43.9)	45 (45.9)	0.83
Number of comorbidities			0.09
0	160 (58.8)	53 (49.5)	
1	75 (27.6)	30 (28.0)	
≥2	37 (13.6)	24 (22.4)	
SOFA score at hospital admission	2 (2 - 3)	3 (3 - 4)	<0.001
Non-respiratory SOFA score at hospital admission	0 (0 - 1)	0 (0 - 1)	0.12
4C mortality score at hospital admission	9 (7 - 11)	11 (8 - 13)	<0.01
4C deterioration score at hospital admission	0.50 (0.40 – 0.61)	0.58 (0.45 – 0.67)	0.001
Variables at hospital admission			
S/F ratio	256 (229 - 300)	204 (120 - 283)	<0.001
FiO_2	0.37 (0.33 - 0.41)	0.45 (0.33 - 0.80)	<0.001
PaO_2 (mmHg)	66 (59 - 75)	73 (60 - 88.50)	0.04
P/F ratio	211 (162 - 272)	153 (112 - 226)	<0.001
Respiratory rate (/min)	24 (20 - 30)	26.50 (22 - 32)	0.01
ROX index	10.4 (8.4 - 14.1)	7.1 (4.6 - 12.7)	<0.001
Variables prior to HFNO start			
S/F ratio	207 (160 - 227)	119 (115 - 121)	<0.001
FiO_2	0.45 (0.41 - 0.60)	0.80 (0.80 - 0.80)	<0.001
Respiratory rate (/min)	26 (24 - 30)	28 (24 - 32)	0.06
ROX index	7.6 (6.1 – 9.3)	4.3 (3.7 – 5.1)	<0.001
Outcome			
Duration of symptoms at hospital admission (days)	8 (7 - 11)	8 (6.3 - 10)	0.74
Time between hospital admission and start HFNO (hours)	8.0 (7.0 – 11.0)	5.0 (2.0 – 56.8)	0.21
Intubation	82 (30.1)	57 (53.3)	<0.001
Intubation ≤4 hours after HFNO start	2 (0.7)	7 (6.5)	<0.01
Intubation ≤6 hours after HFNO start	3 (1.1)	11 (10.3)	<0.001
Intubation ≤2 hours after ICU admission	20 (7.4)	15 (14.0)	0.07
Intubation ≤4 hours after ICU admission	32 (11.8)	24 (22.4)	0.01
In-hospital mortality	9 (3.3)	14 (13.1)	0.001

28-day mortality after HFNO start	5 (1.8)	8 (7.5)	0.02
90-day mortality after HFNO start	10 (3.7)	13 (12.6)	<0.01
Hospital-free days at day 28 after admission (days)	16 (9 - 20)	9 (0 - 16)	<0.001
Hospital-free days at day 60 after admission (days)	48 (41 - 52)	41 (19 - 48)	<0.001
ICU-free days at day 28 after HFNO start (days)	28 (21 - 28)	21 (10 - 28)	<0.001
ICU-free days at day 60 after HFNO start (days)	60 (53 - 60)	53 (42 - 60)	<0.001
Ventilator-free days at day 28 after HFNO start (days)	28 (24 - 28)	24 (13 - 28)	<0.001
Ventilator-free days at day 60 after HFNO start (days)	60 (56 - 60)	56 (45 - 60)	<0.001

Categorical variables are presented as number with percentage, continuous variables are presented as median with Interquartile range. Differences between groups regarding continuous variables were analysed with the Mann–Whitney U test. Differences between groups regarding categorical variables were analysed using the Chi-squared test, or with Fisher’s exact test in case of a cell with less than 5 subjects. Abbreviations: SOFA: Sequential Organ Failure Assessment, HFNO: High-flow Nasal Oxygen, ICU: Intensive Care Unit, S/F ratio: SpO_2-FiO_2 ratio, CRP: C-reactive Protein

Table S10. **Intubated patients, ward- vs. ICU-starters.**

	Ward-starters (n=139)	ICU-starters (n=138)	P-value
Age	63 (54.5 - 70)	62 (54 - 70)	0.81
Male sex	93 (66.9)	104 (75.4)	0.16
Obesity	60 (43.2)	68 (49.3)	0.40
Number of comorbidities			0.64
0	69 (49.6)	63 (45.7)	
1	39 (28.1)	46 (33.3)	
≥2	31 (22.3)	29 (21.0)	
SOFA score at hospital admission	3 (2 – 4)	3 (3 - 3)	0.96
Non-respiratory SOFA score at hospital admission	1 (0 - 1)	0 (0 - 1)	<0.001
4C mortality score at hospital admission	11 (8 - 13)	11 (8 - 13)	0.56
4C deterioration score at hospital admission	0.58 (0.44 - 0.67)	0.61 (0.48 - 0.71)	0.05
Variables at hospital admission			
S/F ratio	232 (157 - 288)	153 (116 - 244)	<0.001
FiO_2	0.41 (0.33 - 0.60)	0.60 (0.38 - 0.80)	<0.001
PaO_2 (mmHg)	69 (62 - 82)	63 (55 - 72)	<0.001
P/F ratio	197 (138 - 262)	110 (81 - 176)	<0.001
Respiratory rate (/min)	25 (22 - 30)	27 (22 – 32)	0.26
ROX index	8.7 (5.7 - 12.7)	5.5 (3.8 - 11.2)	<0.001
Variables prior to HFNO start			
S/F ratio	155 (118 - 206)	115 (111 - 119)	<0.001
FiO_2	0.60 (0.45 - 0.80)	0.80 (0.80 - 0.80)	<0.001
Respiratory rate (/min)	28 (24 - 32)	30 (25 - 35)	<0.01
ROX index	5.3 (4.3 - 7.3)	3.8 (3.3 - 4.6)	<0.001
Laboratory at HFNO start			
CRP (µmol/L)	109 (67.3 - 156)	118 (71.0 - 197)	0.18
Urea (mmol/L)	7.1 (4.9 - 8.8)	6.9 (5.2 - 10.1)	0.26
Ferritin (mg/L)	1060 (463 - 2420)	1240 (618 - 1910)	0.90
Leukocytes (10^9/L)	6.2 (4.5 - 8.3)	8.2 (6.0 - 11.1)	<0.001
Lymphocytes (10^9/L)	0.80 (0.60 - 1.0)	0.80 (0.55 - 1.1)	0.95
Thrombocytes (10^9/L)	195 (157 - 233)	250 (196 - 310)	<0.001

Variables on HFNO prior to intubation			
S/F ratio	95.0 (91.0 - 104.4)	93.4 (89.8 - 100.3)	0.03
P/F ratio	65 (58 - 75)	64 (56 - 73)	0.52
$PaCO_2$ (mmHg)	33.8 (31.0 - 37.0)	35.0 (33.0 - 39.0)	<0.01
Respiratory rate (/min)	30 (26 - 35)	30 (24 - 35)	0.26
ROX index	3.20 (2.76 - 3.76)	3.38 (2.72 - 4.0)	0.41
Outcome			
ICU admission	139 (100)	138 (100)	NA
Time on HFNO until ICU admission (hours)	18.5 (5 – 41)	-	NA
Intubation ≤4 hours after HFNO start	19 (13.8)	9 (6.5)	0.07
Intubation ≤6 hours after HFNO start	29 (21.0)	14 (10.1)	0.02
Intubation ≤2 hours after ICU admission	7 (5.1)	35 (25.2)	<0.001
Intubation ≤4 hours after ICU admission	17 (12.5)	56 (40.3)	<0.001
Time on HFNO until intubation (hours)	37 (14 - 74)	23 (9 – 51)	0.01
Time between ICU admission and intubation (hours)	6 (2 - 22)	23 (9 – 51)	<0.001
Mortality prior to intubation	0	0	NA
Prone positioning after intubation	112 (80.6)	104 (78.2)	0.74
In hospital mortality	23 (16.5)	33 (24.6)	0.13
28-day mortality after HFNO start	13 (9.4)	24 (17.4)	0.06
90-day mortality after HFNO start	22 (15.8)	33 (23.9)	0.09
Hospital-free days at day 28 after admission (days)	0 (0 - 9)	0 (0 - 8)	0.92
Hospital-free days at day 60 after admission (days)	25 (0 - 41)	23.5 (0 - 40)	0.37
ICU-free days at day 28 after HFNO start (days)	10 (1 - 19)	6 (0 - 18)	0.04
ICU-free days at day 60 after HFNO start (days)	42 (14 - 51)	38 (0 - 50)	0.13
Ventilator-free days at day 28 after HFNO start (days)	15 (1 - 22)	12.5 (0 - 21)	0.19
Ventilator-free days at day 60 after HFNO start (days)	47 (15.5 - 54)	42 (0 - 53)	0.16

Categorical variables are presented as number with percentage, continuous variables are presented as median with Interquartile range. Differences between groups regarding continuous variables were analysed Mann–Whitney U test. Differences between groups regarding categorical variables were analysed using the Chi-squared test, or with Fisher's exact test in case of a cell with less than 5 subjects. Abbreviations: SOFA: Sequential Organ Failure Assessment, HFNO: High-flow Nasal Oxygen, ICU: Intensive Care Unit, S/F ratio: SpO_2-FiO_2 ratio, P/F ratio: PaO_2-FiO_2 ratio, CRP: C-reactive Protein

Table S11. **Predictors for ICU admission among patients starting HFNO outside ICU**

**Variable**	**Odds Ratio**	**95% Confidence Interval**	**P-value**
Age	1.01	0.98 - 1.04	0.60
Male sex	1.83	0.95 - 3.60	0.07
Body Mass Index (kg/m^2)	1.00	0.95 - 1.06	0.87
Number of comorbidities			
0	Reference		
1	0.88	0.43 - 1.78	0.72
≥2	0.90	0.36 - 2.20	0.81
Variables prior to HFNO start			
Respiratory rate (/min)	1.09	1.04 - 1.15	0.001
Supplemental oxygen			
Supplemental oxygen group 1	Reference		
Supplemental oxygen group 2	3.35	1.58 - 7.31	0.002
Supplemental oxygen group 3	3.57	1.71 - 7.68	<0.001
SpO_2 prior to start HFNO	0.87	0.78 - 0.96	0.01
Laboratory at HFNO start			
CRP (µmol/L)	1.00	1.00 - 1.00	0.94
Urea (mmol/L)	1.06	0.98 - 1.16	0.17
Leukocytes (10^9/L)	0.94	0.87 - 1.00	0.07
Lymphocytes (10^9/L)	1.03	0.85 - 1.29	0.79
Thrombocytes (10^9/L)	1.00	0.99 - 1.00	0.12
Duration of symptoms at HFNO start (days)	0.88	0.79 - 0.97	0.01

Supplemental oxygen prior to HFNO start was divided into three categories: Group 1: nasal cannula 1-6 L/min or air-entrainment mask 10L/min, Group 2: air-entrainment mask 15L/min or non-rebreathing mask 10 L/min, Group 3: non-rebreathing mask 15L/min. Association between variables and ICU-admission were assessed in a multivariable logistic regression model on all patients who started HFNO outside ICU (n=379, n=186 admitted to ICU).

Abbreviations: HFNO: High-flow Nasal Oxygen, ICU: Intensive Care Unit, SpO_2: oxygen saturation by pulse-oximetry, CRP: C-reactive Protein

References

1. Gupta RK, Harrison EM, Ho A, Docherty AB, Knight SR, van Smeden M, et al. Development and validation of the ISARIC 4C Deterioration model for adults hospitalised with COVID-19: a prospective cohort study. Lancet Respir Med. 2021;9(4):349-59.
2. Grasselli G, Zangrillo A, Zanella A, Antonelli M, Cabrini L, Castelli A, et al. Baseline Characteristics and Outcomes of 1591 Patients Infected With SARS-CoV-2 Admitted to ICUs of the Lombardy Region, Italy. JAMA. 2020;323(16):1574-81.
3. Zhou F, Yu T, Du R, Fan G, Liu Y, Liu Z, et al. Clinical course and risk factors for mortality of adult inpatients with COVID-19 in Wuhan, China: a retrospective cohort study. Lancet. 2020;395(10229):1054-62.
4. Gao M, Piernas C, Astbury NM, Hippisley-Cox J, O'Rahilly S, Aveyard P, et al. Associations between body-mass index and COVID-19 severity in 6.9 million people in England: a prospective, community-based, cohort study. Lancet Diabetes Endocrinol. 2021;9(6):350-9.
5. Williamson EJ, Walker AJ, Bhaskaran K, Bacon S, Bates C, Morton CE, et al. Factors associated with COVID-19-related death using OpenSAFELY. Nature. 2020;584(7821):430-6.
6. Richardson S, Hirsch JS, Narasimhan M, Crawford JM, McGinn T, Davidson KW, et al. Presenting Characteristics, Comorbidities, and Outcomes Among 5700 Patients Hospitalized With COVID-19 in the New York City Area. JAMA. 2020;323(20):2052-9.
7. Mei Y, Weinberg SE, Zhao L, Frink A, Qi C, Behdad A, et al. Risk stratification of hospitalized COVID-19 patients through comparative studies of laboratory results with influenza. EClinicalMedicine. 2020;26:100475.
8. Huang C, Wang Y, Li X, Ren L, Zhao J, Hu Y, et al. Clinical features of patients infected with 2019 novel coronavirus in Wuhan, China. Lancet. 2020;395(10223):497-506.
9. Reyes LF, Murthy S, Garcia-Gallo E, Merson L, Ibanez-Prada ED, Rello J, et al. Respiratory support in patients with severe COVID-19 in the International Severe Acute Respiratory and Emerging Infection (ISARIC) COVID-19 study: a prospective, multinational, observational study. Critical Care (London, England). 2022;26(1):276.
10. Gandhi RT, Lynch JB, Del Rio C. Mild or Moderate Covid-19. N Engl J Med. 2020;383(18):1757-66.
11. Liu L, Xie J, Wu W, Chen H, Li S, He H, et al. A simple nomogram for predicting failure of non-invasive respiratory strategies in adults with COVID-19: a retrospective multicentre study. Lancet Digit Health. 2021;3(3):e166-e74.
12. Xia J, Zhang Y, Ni L, Chen L, Zhou C, Gao C, et al. High-Flow Nasal Oxygen in Coronavirus Disease 2019 Patients With Acute Hypoxemic Respiratory Failure: A Multicenter, Retrospective Cohort Study. Crit Care Med. 2020;48(11):e1079-e86.
13. Mellado-Artigas R, Mujica LE, Ruiz ML, Ferreyro BL, Angriman F, Arruti E, et al. Predictors of failure with high-flow nasal oxygen therapy in COVID-19 patients with acute respiratory failure: a multicenter observational study. J Intensive Care. 2021;9(1):23.
